# Deep Professionalism: Charting a Path for Effective Conflict-of-Interest Management in Medicine

**DOI:** 10.1007/s11606-024-08668-z

**Published:** 2024-02-09

**Authors:** Sunita Sah

**Affiliations:** Cornell Johnson Graduate School of Management, SC Johnson College of Business, Cornell University, Ithaca, NY USA

Conflicts of interest (COIs) threaten the integrity of the medical field due to their capacity to compromise patient trust and healthcare quality. They deserve continued scrutiny but proposed policies to address COIs—ranging from penalties to mandated disclosures—often rest on misguided intuitions about the underlying psychological processes, leading to ineffective or even counterproductive outcomes.^1^

The shortcomings of such policies have ignited calls for a greater focus on intrinsic values.^2^ A defining characteristic of medical professionalism is prioritizing patient well-being over personal interests. Such professionalism, however, may not rectify problems that arise when economic interests sway physicians from placing patients first. Research indicates that a high sense of professionalism can paradoxically contribute to greater *un*ethical behavior.^3^ Through “deep professionalism,” physicians can reach a more accurate understanding of the psychological processes that shape their self-perceptions and engage in greater patient-centric behavior.

## THE FAÇADE OF CLAIMING PROFESSIONALISM

Physicians frequently argue against external controls, such as rules and monitoring, asserting that their professionalism, or intrinsic motivation, ensures ethical conduct and patient welfare.^2^ While a strong sense of professionalism might deter deliberate corruption, it does not shield us from unintentional biases that COIs can introduce. Even worse, an overinflated sense of professionalism can ironically fuel unethical behavior and increase susceptibility to COIs.

This occurs for two reasons. First, professionals who are overconfident in their ability to control bias more readily accept COIs, such as industry gifts and compensation.^3^ This behavior mirrors that of people who, due to strong self-regulation beliefs, keep high-calorie foods or cigarettes nearby, ironically increasing their chances of overeating or smoking. Similarly, a study of 400 managers revealed that professionals with an inflated sense of professionalism were more inclined to accept COIs, mistakenly believing they could resist any unwanted influence.^3^ The most effective safeguard against COIs is to reject them whenever possible. However, those who believe that their professionalism will help them self-regulate and manage unwanted influence are less likely to do so.

Second, when physicians accept COIs, a strong sense of professionalism can result in increased bias in medical decisions. Several studies on cognitive bias confirm that individuals who perceive themselves as unbiased are more likely to act in biased ways. For instance, in organizations that emphasize meritocracy, managers often display greater bias against women in evaluations and career advancements;^4^ similarly, people with strong beliefs in their objectivity demonstrate increased bias in hiring decisions.^5^ Simply put, confidence in one’s impartiality can facilitate bias, by leading individuals to believe their preferences reflect objective reality. Similarly, a high self-perception of professionalism can lead to less self-scrutiny and correction for undue influence, and thus greater bias.^3^ Given that many medical COIs are unavoidable, this is an important concern. The majority of US physicians practice in environments in which the services they provide directly affect their income, the most prominent being the fee-for-service model that leads to distortions of patient care.

Taken together, claims of “professionalism,” in its impoverished, shallow, and ill-understood form, can inadvertently lead to greater acceptance of COIs and increased bias.^3^ Alarmingly, people with a high self-concept of professionalism cannot predict their bias in advance nor recognize it in hindsight.

## DEEP PROFESSIONALISM

With the concept of “deep professionalism,” physicians can move beyond simply claiming confidence in their ethical decisions to actually making themselves more resistant to COIs.^3^ Deep professionalism demands two elements: (1) a full understanding of the limitations of self-regulation gained via reflection, cognitive moral development, and intellectual humility; and (2) a consistent practice of behaviors embodying this understanding to serve patients and society.

Recognizing one’s fallibility is crucial for deep professionalism. Given the complexity and uncertainty of medical decisions, it is easy for physicians to convince themselves that their choices always prioritize patients’ interests, particularly with COIs, where biases (or failures) that result from them are unseen. The heterogeneity of treatment decisions makes it impossible to determine bias at an individual physician level. While the “see one, do one, teach one” approach in medicine can foster technical skill development and decisive action, primarily because success or failure is often immediately apparent, the lack of feedback on influence from COIs can spur overconfidence in one’s ability to manage them and a lack of ability to recognize failures. In these more ambiguous situations, we need intellectual humility to avoid the double curse of lacking skill and failing to recognize our lack of skill.

Education on self-regulation is insufficient on its own, as it often leads to the belief that everyone is biased, except oneself.^1^ Intellectual humility requires recognizing the *limits* of one’s knowledge and engaging in work and reflection to overcome the cognitive biases that distort self-appraisal.^6^ For instance, documenting “known unknowns” increases intellectual humility, which can protect against human errors and bias, and enhances learning and decision-making.^6^ Accordingly, instead of asserting that financial ties do not influence them, physicians could state, “It is unknown how much I’ve been influenced.” This type of self-doubt is necessary to put patients first in domains of unwanted influence.

Reflecting Aristotle’s assertion that virtues of character are honed through repetition, deep professionalism also requires habitual dedication to prioritizing patients over self-interest. For example, if an institution bans gifts over $100, deep professionals comprehend that even smaller gifts can wield undue influence. Hence, they refuse all gifts, not merely adhering to the letter of the law but consistently living the spirit behind it.

Deep professionalism transcends a self-perceived intrinsic attribute; it is a deep-seated understanding of one’s limitations, married with consistent ethical behavior. If not embraced in this manner, professionalism risks being merely a veneer, a self-serving ideology that exacerbates rather than resolves issues.

## A MULTI-DIMENSIONAL APPROACH FOR ETHICAL PRACTICE

Although external policies have limitations, relying solely on subjective intrinsic feelings can also fail and have a contrary effect. To effectively manage COIs, we need to combine both effective policies with deep professionalism.

First, structural reforms can support the practice of deep professionalism by aligning provider incentives with patient interests. Paying physicians by salary reduces some conflicts, but others, such as conflicts between patient and insurer interests, may remain. Reminding physicians to place patients first decreases bias from COIs, but not to the same extent as eliminating conflicts completely. Reducing industry conflicts is relatively easier: some medical centers have already led the way, prohibiting physicians from accepting industry gifts, resulting in less bias.^7^

Second, we need to bolster educational initiatives and practices to enhance understanding and management of COIs. When structural reforms are lacking (and rules are never exhaustive), deep professionalism provides some protection of ethical values. Comprehensive medical training should not only illuminate the existence of conflicts but also equip individuals to recognize, articulate, and strategize against potential adverse influences. As intellectual humility is situation-dependent,^6^ deep professionalism needs to be cultivated as a *practice* not a *character trait*—one reinforced by leaders that exemplify such ethical behavior.

Although external policies and intrinsic values serve a purpose, they are insufficient on their own. People draw on multiple sources of information to perceive norms, including leaders, peers, and institutional/legal signals such as sanctions or other penalties and rewards. To uphold the integrity of the medical profession, a holistic, multi-faceted approach that effectively combines structural reforms with the cultivation of deep professionalism is absolutely necessary.
